# THE RELATIONSHIP OF APOE ε4 TO THE RELATIVE TIMES AND HAZARDS OF DEMENTIA

**DOI:** 10.1093/geroni/igz038.2175

**Published:** 2019-11-08

**Authors:** Danielle Powell, Pei-Lun Kuo, Jennifer A Deal, Rebecca Gottesman, Priya Palta, David Knopman, Alden L Gross

**Affiliations:** 1 Johns Hopkins University Bloomberg School of Public Health, Baltimore, Maryland, United States; 3 Johns Hopkins University, Baltimore, Maryland, United States; 4 Columbia University, New York, New York, United States; 5 Mayo Clinic, Rochester, Minnesota, United States; 6 Johns Hopkins Bloomberg School of Public Health, Baltimore, Maryland, United States

## Abstract

Although APOE ε4 is an established risk factor for dementia, it is unclear whether it is associated with elevated risk or earlier symptom onset. Longitudinal study of 10,400 white-race individuals in the ARIC cohort followed from age 60 to incident dementia diagnosis, death, or censoring. All-cause dementia was defined using standardized algorithms incorporating longitudinal cognitive change, proxy report, and hospital or death certificate dementia codes. Death was ascertained via the National Death Index and death certificates. We used a parametric mixture of generalized gamma distributions to simultaneously estimate the distribution of event times and the proportion of individuals who experience each outcome (i.e., dementia and its competing risk, dementia-free death) by APOE ε4 status (≥ 1 allele vs. no alleles). Age-adjustment was through use of age as the time scale. APOE ε4 carrier status was associated with a doubling of the overall frequency of dementia incidence to 25% compared to 13% (p < 0.001) in non- ε4 carriers. The distributions of time to dementia was modified by APOE ε4 status (p=0.007): median time to dementia onset among APOE ε4 carriers was 81.9 years compared to 83.3 years in non-APOE ε4 carriers (p = 0.005). No differences in results were found by sex. APOE ε4 carrier status is associated with both elevated risk and earlier time to dementia onset. These findings clarify the causal role of APOE in dementia etiology, could help better identify at-risk subgroups, and may help facilitate better research recruitment.

